# Importance of optimal dosing ≥30 mg/kg/d during deferasirox treatment: 2.7-yr follow-up from the ESCALATOR study in patients with β-thalassaemia

**DOI:** 10.1111/j.1600-0609.2011.01662.x

**Published:** 2011-10

**Authors:** Ali Taher, Mohsen S Elalfy, Kusai Al Zir, Shahina Daar, Abdullah Al Jefri, Dany Habr, Ulrike Kriemler-Krahn, Ali El-Ali, Bernard Roubert, Amal El-Beshlawy

**Affiliations:** 1 American University of Beirut Medical CenterBeirut, Lebanon; 2 Ain Shams UniversityCairo, Egypt; 3 National Thalassemia CenterDamascus, Syrian Arab Republic; 4 Sultan Qaboos UniversityMuscat, Oman; 5 King Faisal Specialist Hospital & Research CenterRiyadh, Saudi Arabia; 6 Novartis PharmaceuticalsEast Hanover, NJ, USA; 7 Novartis Pharma AGBasel, Switzerland; 8 Cairo UniversityCairo, Egypt

**Keywords:** deferasirox, iron chelation therapy, iron overload, β-thalassaemia, efficacy

## Abstract

Following 1-yr deferasirox therapy in the ESCALATOR study, 57% of previously chelated patients with β-thalassaemia achieved treatment success (maintenance of or reduction in liver iron concentration (LIC) vs. baseline LIC). Seventy-eight per cent had dose increases at median of 26 wk, suggesting that 1-yr results may not have reflected full deferasirox efficacy. Extension data are presented here. Deferasirox starting dose was 20 mg/kg/d (increases to 30/40 mg/kg/d permitted in the core/extension, respectively). Efficacy was primarily assessed by absolute change in LIC and serum ferritin. Overall, 231 patients received deferasirox in the extension; 67.4% (*P* < 0.0001) achieved treatment success. By the end of the extension, 66.2% of patients were receiving doses ≥30 mg/kg/d. By the end of the 1-yr extension, mean LIC had decreased by 6.6 ± 9.4 mg Fe/g dw (baseline 19.6 ± 9.2; *P* < 0.001) and median serum ferritin by 929 ng/mL (baseline 3356; *P* < 0.0001). There was a concomitant improvement in liver function markers (*P* < 0.0001). Fewer drug-related adverse events were reported in extension than core study (23.8% vs. 44.3%). Doses ≥30 mg/kg/d were generally required because of high transfusional iron intake and high baseline serum ferritin levels, highlighting the importance of administering an adequate dose to achieve net negative iron balance.

Iron chelation therapy is essential in patients with β-thalassaemia major undergoing regular transfusion therapy to potentially prevent the serious clinical sequelae of transfusional iron overload, such as cardiac failure. Evaluation of the long-term effect of iron chelation in paediatric patients with thalassaemia is of particular importance because of the known negative impact on growth and development of iron toxicity to endocrine organs (1–3) and adverse effects of deferoxamine (DFO, Desferal®) treatment (4, 5).

Previous studies with the once-daily oral iron chelator deferasirox (Exjade®) have demonstrated that dosing should be guided by the patient's current iron burden and ongoing transfusional iron intake (6–8). The ESCALATOR (Efficacy and Safety of long-term treatment with ICL670 in β-thALAssaemia patients with Transfusional hemOsideRosis) study was designed to evaluate the efficacy of deferasirox initiated at a dose of 20 mg/kg/d, followed by dose adjustments in response to efficacy and safety markers. The study enrolled heavily iron-overloaded patients with β-thalassaemia major, all of whom were previously unsuccessfully chelated with DFO and/or deferiprone (Ferriprox®) (9). Following 1-yr treatment with deferasirox, 57% of patients achieved treatment success (maintenance of or reduction in LIC depending on baseline LIC). To achieve these therapeutic goals, 78% of patients required dose increases above the 20 mg/kg/d starting dose; however, most of these patients did not receive a dose increase to ≥25 mg/kg/d until more than halfway through the 1-yr study (9). Therefore, the effect of deferasirox on LIC and serum ferritin levels after 1 yr of treatment may not have fully reflected the efficacy of this dosing strategy because patients received insufficient chelation to achieve net iron reduction while being transfused on an ongoing basis. In this study, we report the efficacy and safety of deferasirox during the ESCALATOR extension study, which collected data for at least 1 additional year of treatment.

## Methods

### Study design and patient recruitment

The ESCALATOR study was a prospective, open-label multicentre trial conducted at seven sites in the Middle East; data reported in this study are acquired from patients from six sites (9). The seventh site was excluded for a good clinical practice (GCP) reason. Patients aged ≥2 yr with β-thalassaemia and transfusional iron overload (LIC ≥2 mg Fe/g dry weight [dw] and serum ferritin ≥500 ng/mL) were recruited to the 1-yr core study; all enrolled patients had previously been suboptimally chelated with DFO and/or deferiprone. Paediatric patients were classified as aged 2–<16 yr and adults as aged ≥16 yr. Patients who completed the core study were eligible for inclusion in the extension study, which lasted for an additional 2 yr or until deferasirox became commercially available locally, whichever came first. Patients who were taken off the study for the latter reason were considered to have completed treatment. All patients (or parents/guardians) provided written, informed consent, and the study was conducted in accordance with GCP guidelines and the Declaration of Helsinki.

### Dose selection

In the 1-yr core study, all patients started treatment with deferasirox 20 mg/kg/d, except for three patients who received an initial dose of 10 mg/kg/d. This dose was subsequently increased to 20 mg/kg/d based on protocol amendment. Dose adjustments in the range 0–30 mg/kg/d were permitted in the core and extension studies. Deferasirox dose at the start of the extension study was based on the final dose in the core study. Throughout the core and extension studies, routine dose adjustments in the increments of 5 or 10 mg/kg/d were performed based on efficacy (serum ferritin trends) and safety markers. Following a protocol amendment, dose increases above 30 mg/kg/d were permitted from the start of the extension study (total dose range 0–40 mg/kg/d). Doses were increased if serum ferritin levels increased by ≥1000 ng/mL above baseline on two consecutive visits or were >2500 ng/mL (changed to >1000 ng/mL in amendment 4) without a decreasing trend (or >500 ng/mL with an increasing trend, added in amendment 4). Deferasirox administration was interrupted if serum ferritin levels fell to ≤500 ng/mL on two consecutive visits and subsequently resumed if serum ferritin levels rose to ≥1000 ng/mL (changed to >500 ng/mL in amendment 4). Amendment 4 of the protocol was introduced in the extension study and affected only a few patients. Doses were reduced if the patient displayed elevated levels of serum creatinine, urinary protein/creatinine ratio and transaminases, and in response to adverse events (AEs).

### Assessments

The primary objectives during the extension study were to allow patients treated with deferasirox during the core to continue on study drug and to monitor the long-term safety and tolerability of deferasirox. The primary efficacy endpoint of the study was treatment success, defined as a reduction in LIC of ≥3 mg Fe/g dw if baseline LIC was ≥10 mg Fe/g dw, or final LIC of 1–<7 mg Fe/g dw if core baseline LIC was 1–<7 or ≥7–<10 mg Fe/g dw. This success definition reflects the treatment goals of maintenance or reduction of iron burden according to the patient's baseline status. Secondary efficacy endpoints were absolute change from core baseline in LIC and serum ferritin levels. LIC was determined by biopsy for all patients at core baseline and after 1 yr of treatment at the end of the core study; all measurements were performed in a central laboratory using atomic absorption spectrometry and were performed according to standardised procedures. In the extension study, LIC was determined by R2 magnetic resonance imaging (MRI) for all patients at extension baseline, after 1 yr of treatment and at the end of the extension study. LIC was assessed by MRI measurements of the proton transverse relaxation parameter R2 using 5-mm axial slices. R2 scans were performed using FerriScan® technology (Resonance Health) (10). Serum ferritin levels were assessed at baseline and every 4 wk as a potential surrogate marker for LIC.

Safety and tolerability were also evaluated across the study period by monitoring the incidence and type of AEs and by assessing routine laboratory parameters. Left ventricular ejection fraction (LVEF) was assessed using 2-dimensional echocardiography at rest and on exertion at core baseline and at 6-month intervals throughout the core and the extension studies. To ensure consistency, the same cardiologist at each participating centre interpreted all echocardiograms. Compliance was assessed by means of returned tablet counts. Paediatric assessments of growth were performed based on change in height, which was measured using a Harpenden Stadiometer (11) at core baseline and regularly throughout treatment and expressed as height standard deviation scores (h-SDS). SDS are standardised normal distributed scores with mean 0 and standard deviation 1 of an age- and gender-specific normal population. Pubertal stage (female breast development and male testes volume) was scored every 3 months with a physical examination according to the Tanner staging system (12, 13).

### Statistical methods

The statistical methods used to analyse efficacy in the extension study were as previously described for the core study (9). The intent-to-treat efficacy analysis included all patients enrolled in the extension study. Efficacy data are primarily presented for the first 2 yr of deferasirox treatment only, i.e. the 1-yr core study and year 1 of the extension study. This is because the protocol mandated that patients were to be taken off the study once deferasirox became commercially available in their country; as such, patients received study drug for a very variable duration and only a small number completed year 2 of the extension although they continued to receive deferasirox outside of the trial. In addition, because patients were not dose escalated to ≥30 mg/kg/d until after the core study, efficacy data stratified by mean actual dose (<30 and ≥30 mg/kg/d) were only calculated and presented during year 1 of the extension study. This was to ensure that there were a sufficient number of patients in the higher-dose cohort from which to draw more robust efficacy conclusions.

All patients who received at least one dose of study medication in the extension study were included in the safety population. Safety data are presented for the entire treatment period, i.e. including year 2 of the extension study. Safety data stratified by mean actual dose (<30 and ≥30 mg/kg/d) were therefore calculated and presented from baseline in the core study.

## Results

### Patient characteristics

Overall, 237 patients [162 paediatric patients (aged 2–<16 yr) and 75 adults (aged ≥ 16 yr)] were included in the core study and 233 (162 paediatric patients and 71 adult patients) completed 1 yr of treatment; 231 of these patients entered year 1 of the extension study and received deferasirox (Table 1). There were 162 paediatric patients and 69 adults. Two additional adult patients entered year 1 of the extension study, but did not receive study drug because serum ferritin levels dropped below the protocol-specified target of 500 ng/mL and did not subsequently increase to above 1000 ng/mL. In total, 167 patients (120 paediatric patients and 47 adult patients) entered year 2 of the extension study.

**Table 1:** Demographic and baseline characteristics of patients who received ≥1 dose of deferasirox during the extension study

	Paediatric patients (*n* = 162)	Adult patients (*n* = 69)
Mean age ± SD, yr	9.5 ± 3.6	21.2 ± 5.8
Female/male, *n*	80:82	34:35
Race (Caucasian/Oriental/other), *n*	59:82:21	11:38:20
History of hepatitis B only, *n* (%)	0 (0)	0 (0)
History of hepatitis C only, *n* (%)	41 (25.3)	26 (37.7)
History of hepatitis B and C, *n* (%)	3 (1.9)	1 (1.4)
Splenectomy, *n* (%)	46 (28.4)	47 (68.1)
Previous chelation therapy, *n* (%)		
Deferoxamine (DFO) monotherapy	145 (89.5)	39 (56.5)
Deferiprone monotherapy	1 (0.6)	3 (4.3)
DFO + deferiprone^1^	16 (9.9)	27 (39.1)
Median duration of previous chelation therapy (range), yr^2^	5.2 (0.1–13.2)	12.2 (3.1–21.0)
Mean transfusion history duration ± SD, yr	8.6 ± 3.7	18.9 ± 5.6
Mean number of transfusion sessions in the year prior to study entry ± SD	15.5 ± 4.4	14.3 ± 3.8
Mean amount transfused in the year prior to study entry ± SD, mL/kg/d	0.50 ± 0.18	0.39 ± 0.17

SD, standard deviation.

1 Patients had received prior chelation with DFO and deferiprone, although these may not have been given in combination.

2 Paediatric patients *n* = 121 and adult patients *n* = 49.

### Deferasirox dosing

Overall, patients received deferasirox for a median duration of 140 wk (range 59–161; 2.7 yr). In the 1-yr core study, overall mean actual deferasirox dose was 22.2 ± 3.5 mg/kg/d (21.6 ± 3.3 and 24.0 ± 3.4 mg/kg/d in paediatric and adult patients, respectively). In the extension study, over a median period of 1.7 yr, mean actual deferasirox dose was 27.1 ± 5.6 mg/kg/d (26.8 ± 5.2 and 27.9 ± 6.6 mg/kg/d in paediatric and adult patients, respectively). When dosing was evaluated during year 1 of the extension study only, 59 patients (25.5%) received average actual deferasirox doses ≥30 mg/kg/d (mean 32.2 ± 1.6); 172 (74.5%) received actual doses of <30 mg/kg/d (mean 24.6 ± 4.4).

Overall, 206 of 231 patients (89.2%) had at least one dose increase during the core or extension study, which occurred after a median of 38 wk (range 3–132). Time to first dose increase was longer in paediatric (40 wk, range 3–132) than in adult patients (26 wk, range 4–116). The most common dose increase was from 20 to 30 mg/kg/d (146 patients) during the core and extension. In the extension study, 140 patients (60.6%) had at least one dose increase; median time to increase was comparable among paediatric (35 wk, range 3–89) and adult patients (33 wk, range 4–81). During the extension study, six patients (2.6%) had dose decreases and 13 patients (5.6%) had dose interruptions because of AEs or abnormal laboratory values. Dose decreases or temporary interruptions occurred in four (1.7%) and 15 (6.5%) patients, respectively, who achieved their therapeutic goal. At the end of the extension study, the final deferasirox dose was ≥30 mg/kg/d in 153 patients [66.2%; 112 paediatric patients (69.1%) and 41 adult patients (59.4%)]. During the core and extension studies, 220 patients (95.2%) were >90% compliant with deferasirox therapy.

### Transfusional iron intake

Over the course of the 2.7-yr study, mean transfusional iron intake in patients who completed the study was 0.33 ± 0.09 mg/kg/d and iron intake was significantly higher in paediatric (0.35 ± 0.09 mg/kg/d; *n* = 120) than in adult patients (0.28 ± 0.08 mg/kg/d; *n* = 41) (*P* < 0.0001). Transfusional iron intake was also different across deferasirox dose cohorts (0.32 ± 0.09 mg/kg/d for <30 mg/kg/d cohort and 0.40 ± 0.10 mg/kg/d for ≥30 mg/kg/d cohort [*P* = 0.0008]), reflecting the need for higher doses in response to higher ongoing iron intake.

### Efficacy

*Treatment success and overall efficacy.* Overall treatment success rate from core baseline to the end of year 1 of the extension study was 67.4% (95% confidence intervals [CI] 61.4–73.4%; *P* < 0.0001). In the paediatric and adult populations, treatment success rate was 63.0% (95% CI 55.5–70.4%) and 77.5% (95% CI 67.8–87.2), respectively. Of 69 patients with baseline serum ferritin levels <2500 ng/mL, 64 (92.8%) of these patients maintained a serum ferritin level <2500 ng/mL by the end of the 1-yr extension and experienced a mean decrease in LIC of 8.1 ± 7.4 mg Fe/g dw; five (7.2%) patients progressed to ≥2500 ng/mL by the end of the 1-yr extension with a mean increase in LIC of 6.5 ± 5.3 mg Fe/g dw. Of 142 patients with baseline serum ferritin levels ≥2500 ng/mL, 90 (63.4%) patients maintained this level by the end of the 1-yr extension and experienced a mean reduction in LIC of 2.4 ± 9.8 mg Fe/g dw; 52 (36.6%) patients progressed to serum ferritin levels <2500 ng/mL by the end of the 1-yr extension with a mean reduction in LIC of 13.4 ± 5.6 mg Fe/g dw.

**Figure 1:**
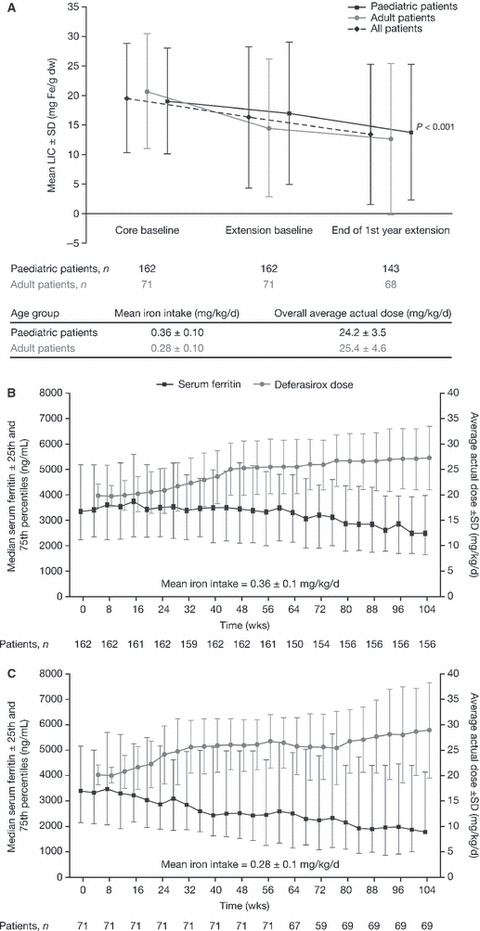
(A) Mean change in liver iron concentration ± SD from core baseline to end of year 1 of the extension in paediatric, adult and all patients. Mean actual deferasirox ± SD dose and median serum ferritin levels ± 25th/75th percentiles from core baseline to end of year 1 of the extension in (B) paediatric and (C) adult patients. Note: iron intake values shown in (A) are from the core baseline until the end of the 1-yr extension.

Overall mean LIC decreased from 19.6 ± 9.2 mg Fe/g dw at core baseline to 13.4 ± 11.9 mg Fe/g dw by the end of the 1-yr extension (Fig. 1A), and this represents a significant mean decrease of 6.6 ± 9.4 mg Fe/g dw (*P* < 0.001; relative change −32.4%). At core baseline, 33.9% of patients (79/233) had LIC <15 mg Fe/g dw, which increased to 55.8% (130/233) at extension baseline and 67.8% (143/211) at the end of year 1 of the extension. In these patients where the serum ferritin level reached <2500 ng/mL by the end of the 1-yr extension, mean LIC decreased by 10.5 ± 7.2 mg Fe/g dw; in those with serum ferritin level ≥2500 ng/mL at the end of the 1-yr extension, mean LIC decreased by 1.9 ± 9.8 mg Fe/g dw. Serum ferritin levels decreased steadily in the entire population during the core and extension studies, with a median reduction of 929 ng/mL (range −7978 to −3983, *P* < 0.0001; relative change −30.2%) by the end of year 1 of the extension. Decreases in serum ferritin levels were reflective of dose escalation in the overall population. Overall, 32.6% (76/233), 40.3% (94/233) and 54.2% (122/225) of patients had serum ferritin levels of <2500 ng/mL at core baseline, extension baseline and end of year 1 of the extension, respectively.

*Efficacy in paediatric and adult patients.* The reduction in LIC over the initial 2-yr treatment period was 5.7 ± 9.1 mg Fe/g dw in paediatric patients (19.1 ± 9.0 to 13.8 ± 11.5) and 8.5 ± 9.9 mg Fe/g dw (20.7 ± 9.8–12.6 ± 12.8) in adults (*P <*0.0001 for both, Fig. 1A). Decreases in serum ferritin levels over the same period were reflective of dose escalation in both paediatric and adult patients (Fig. 1B,C). The overall reduction in serum ferritin levels from core baseline to the end of year 1 of the extension was 853 ng/mL (3326–2474) in paediatric patients and 1254 ng/mL (3396–1776) in adult patients (*P* < 0.0001 for both).

**Figure 2:**
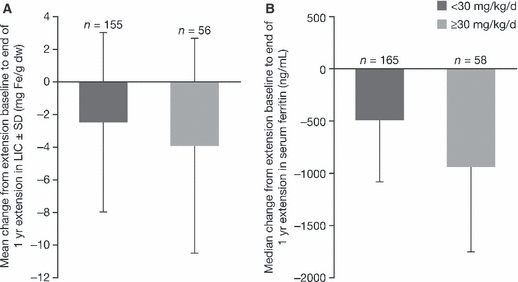
(A) Mean absolute change in liver iron concentration ± SD and (B) median absolute change in serum ferritin levels ± 25th and 75th percentiles from extension baseline to end of year 1 of the extension in patients who received mean actual deferasirox doses of <30 or ≥30 mg/kg/d.

*Efficacy by mean actual dose groups.* In patients who received an average actual dose of ≥30 mg/kg/d in the 1-yr extension, LIC decreased significantly by 3.9 ± 6.6 mg Fe/g dw (*P* < 0.0001). In patients who received an actual dose of <30 mg/kg/d mean LIC decreased by 2.5 ± 5.5 mg Fe/g dw (*P* < 0.0001) (Fig. 2A). A comparison of relative percentage change in LIC in the 1-yr extension between the ≥30 mg/kg/d and <30 mg/kg/d groups revealed that this change was not significantly different between both cohorts (*P* = 0.932). In patients who received an average actual dose of ≥30 mg/kg/d, the median decrease in serum ferritin levels during year 1 of the extension was significant at 937 ng/mL (*P* < 0.0001); in patients who received an average actual dose of <30 mg/kg/d, there was a significant median decrease of 493 ng/mL (*P* < 0.0001) (Fig. 2B). A comparison of relative percentage change in serum ferritin levels in the 1-yr extension between the ≥30 mg/kg/d and <30 mg/kg/d groups also revealed that this change was not significantly different between these two groups (*P* = 0.832).

*Efficacy during year 2 of the extension.* In total, 167 patients entered year 2 of the extension study. The median duration of exposure in year 2 of the extension was 276 d, and a total of 51 patients had serum ferritin assessments at 36 months. During this period, the number of patients remaining in the study declined; patients received deferasirox for varying durations that were related to the commercial availability of deferasirox in each participating country. Mean LIC at the start of year 2 of the extension was 15.6 ± 11.9 mg Fe/g dw, with a mean decrease of 3.0 ± 5.7 mg Fe/g dw (*P* < 0.0001) from this point to the end of the study. Median serum ferritin decreased by 213 ng/mL during the same period (last observation carried forward at the median exposure), from 2851 ng/mL at the start of year 2 of the extension.

### Safety and tolerability

Overall, 216 patients (92.7%) completed the extension study; overall median exposure to study drug was 88.1 wk (1.7 yr) during the extension. Of the 17 discontinuations, 10 and seven occurred in years 1 and 2 of the extension, respectively. Reasons for discontinuation were as follows: loss to follow-up (*n* = 8, 3.4%), AEs (*n* = 3, 1.3%), death (*n* = 3, 1.3%), protocol violation (*n* = 2, 0.9%) and consent withdrawal (*n* = 1, 0.4%). Of the three deaths reported during the extension study, one was a paediatric patient (as a result of respiratory failure) and two were adults (as a result of cerebral and subarachnoid haemorrhage); none were considered to be related to study drug. Of the three AEs that led to discontinuation, only one (abdominal discomfort in an adult) was considered by the investigators to be drug related and the other two AEs were cardiac failure and hypotension (in a paediatric and adult patient, respectively). This single incidence of cardiac failure was related to progression of the underlying disease.

During the core and extension studies, AEs regardless of relationship to study drug were reported by 201 (87.0%) patients. AEs that were considered by the investigators to be drug related were noted in 126 patients (54.5%), and most (>95%) were mild to moderate in nature. The most commonly reported drug-related AEs are summarised in Table 2. The overall pattern and frequency of AEs were generally comparable between the paediatric and adult populations; however, of the most commonly reported AEs, nausea and increased serum creatinine levels were observed more frequently in adult patients, while vomiting and increased alanine aminotransferase (ALT) were noted more frequently in paediatric patients. Fifty-five patients (23.8%) reported drug-related AEs in the extension study, compared with 105 patients (44.3%) solely in the core study (9).

**Table 2:** Most common (>3%) drug-related adverse events (AEs)

AE	Overall (*n* = 231)	Core^1^ (*n* = 237)	Extension (*n* = 231)	Paediatric patients (*n* = 162)	Adult patients (*n* = 69)	<30 mg/kg (*n* = 204)	≥30 mg/kg (*n* = 27)
Median deferasirox exposure, weeks	140.1	52.3	88.1	143.9	122.3	139.4	155.0
Nausea	16 (6.9)	17 (7.2)	4 (1.7)	6 (3.7)	10 (14.4)	11 (5.4)	5 (18.5)
Vomiting	28 (12.1)	21 (8.9)	14 (6.1)	21 (13.0)	7 (10.1)	24 (11.8)	4 (14.8)
Increased ALT^2^	23 (10.0)	13 (5.5)	16 (6.9)	18 (11.1)	5 (7.2)	20 (9.8)	3 (11.1)
Increased serum creatinine^2^	23 (10.0)	9 (3.8)	14 (6.1)	8 (4.9)	15 (21.7)	20 (9.8)	3 (11.1)
Rash	20 (8.7)	19 (8.0)	2 (0.9)	13 (8.0)	7 (10.1)	16 (7.8)	4 (14.8)

ALT, alanine aminotransferase.

1 Ref. 9.

2 Reported as an AE by the investigator.

Serious AEs were experienced by 26 patients (11.3%). The most common were upper abdominal pain (*n* = 3, 1.3%), upper limb fractures (*n* = 3, 1.3%), vomiting (*n* = 3, 1.3%) and cholecystitis (*n* = 3, 1.3%). Three serious AEs were considered to be related to the study drug: severe peptic ulcer perforation (*n* = 1), moderate increased serum creatinine levels (*n* = 1) and moderate cholestatic jaundice (*n* = 1).

**Figure 3:**
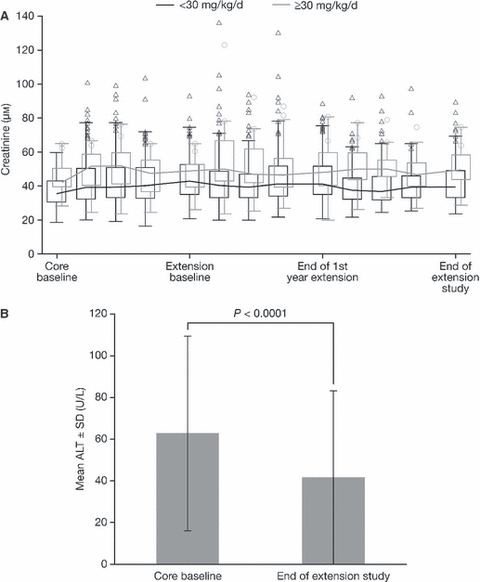
(A) Serum creatinine levels stratified by dose category. Note: The boxes represent the 25th and 75th percentiles, while the whiskers correspond to the 10th and 90th percentiles. The medians are connected. Outliers ≥95% are indicated as individual points. (B) ALT levels at core baseline and at the end of extension study.

*Renal and hepatic function.* Eight patients (3.5%) had two consecutive serum creatinine level increases >33% above core baseline and greater than upper limit of normal (ULN) in the core and extension studies; six of these were paediatric patients and two were adult patients. All eight patients continued to receive deferasirox during the core and extension studies. Creatinine levels had returned to within the normal range at the last assessment performed in six (four paediatric and two adult) of these patients. One adult patient of these eight had multiple serious AEs including cholecystitis, elevated serum creatinine levels, congestive cardiac failure and arrhythmia, although all these events resolved. Overall mean serum creatinine levels remained stable over the core and extension studies, irrespective of average actual deferasirox dose received (Fig. 3A).

Eight patients (3.5%), six paediatric and two adult, had two consecutive increases in ALT > 10 × ULN, and baseline levels were already elevated in five patients. ALT levels at the last assessment were below baseline in five of the eight patients. Two of the remaining three patients had normal levels at baseline; therefore, only one patient with elevated baseline ALT still had elevated levels at the last assessment and this patient continued to receive deferasirox. One patient of these eight discontinued with deferasirox.

The mean level of ALT at core baseline was 62.7 ± 46.6 U/L (*n* = 230) and had decreased to 41.7 ± 40.9 U/L (*n* = 181) at the end of the extension study, representing an overall significant decrease of 24.8 ± 48.2 U/L (*P* < 0.0001; relative change −5.8 ± 137.1%, Fig. 3B). Mean aspartate aminotransferase (AST) levels decreased significantly from 50.4 ± 33.9 U/L (*n* = 230) to 41.0 ± 30.3 U/L (*n* = 181) over the same treatment period, and overall mean decrease was 11.7 ± 27.7 U/L (*P* < 0.0001; relative change −11.5 ± 60.3%).

*Cardiac function.* During deferasirox treatment, there was a statistically significant increase in mean LVEF from 65.2 ± 6.8% at core baseline to 68.2 ± 7.8% (*n* = 171) at the end of the extension study (mean increase 2.3 ± 8.6% and *P* = 0.0007).

**Figure 4:**
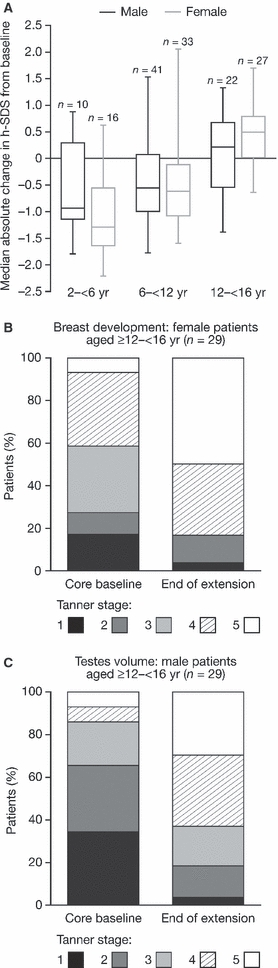
(A) Median absolute change in h-SDS ± 25th and 75th percentiles for male and female paediatric patients during the core and extension study. Whiskers represent maximum and minimum values. (B) Tanner stage assessments of breast development in females and (C) testes volume in males.

*Paediatric growth and development.* Overall, 162 paediatric patients (aged 2–<16) entered the extension study. As previously reported, at core baseline males and females in all age groups were shorter than those in the Center for Disease Control reference population (9). Steady increases in height were noted for paediatric patients aged 2–<16 over the treatment period. Absolute change for h-SDS indicated that the growth of paediatric patients predominantly followed the normal trends derived from US clinical growth charts (Fig. 4A). Sexual development in patients aged ≥12–<16 yr, as evaluated by Tanner stage assessments, also progressed normally during the course of deferasirox treatment and was comparable with a US reference population (14). The proportion of patients at Tanner stage 5 for female breast development increased from 6.9% at core baseline to 41.4% at the end of the entire extension (Fig. 4B). At core baseline, 6.9% of male patients were at Tanner stage 5 for testicular volume, which increased to 27.6% by the end of the entire extension (Fig. 4C).

## Discussion

ESCALATOR was the first study to evaluate whether a fixed starting dose of deferasirox 20 mg/kg/d, followed by dose titration based on iron burden and transfusional iron intake, could provide effective iron chelation in heavily iron-overloaded patients with β-thalassaemia major (9). By the end of the core and extension studies, patients’ overall LIC and serum ferritin levels were significantly reduced from baseline. In addition, based on prospectively defined criteria, a statistically significant proportion of patients achieved treatment success (67.4%; *P* < 0.0001) vs. 57% at the end of the core study. It should be noted that time to first dose increase in the core study was later than required for effective iron chelation. Dose increases to ≥30 mg/kg/d were generally required in this population of heavily transfused, iron-overloaded patients, and final deferasirox dose at the end of the extension study was ≥30 mg/kg/d in 66% of patients. These findings are consistent with earlier deferasirox studies, which found that ≤20 mg/kg/d is not sufficient to significantly reduce iron burden in heavily iron-overloaded patients with thalassaemia (6, 15, 16). The recommended initial daily starting dose of deferasirox is 20 mg/kg/d for most patients. For patients with a higher transfusional iron intake (e.g > 14 mL/kg/month packed red blood cells for adults), a starting dose of 30 mg/kg/d is recommended. For patients experiencing poor control of iron overload at doses up to 30 mg/kg/d, dosing up to a maximum of 40 mg/kg/d may be required (17). Compared with patients who received an average actual deferasirox dose of <30 mg/kg/d, those who received ≥30 mg/kg/d during the extension had higher LIC and serum ferritin levels at extension baseline and subsequently achieved greater decreases in these iron parameters at the end of year 1 of the extension despite a higher transfusional iron intake (0.40 ± 0.10 mg/kg/d vs. 0.32 ± 0.09 mg/kg/d). This observation highlights that timely dose increases above 30 mg/kg/d are necessary for effective reduction of iron in patients with heavy iron burden and high iron intake. Thus, patients with heavy iron overload may require rapid dose titration to meet therapeutic goals of iron reduction. The results presented here support the concept of higher initial starting doses followed by dose titrations.

Substantial decreases in serum ferritin levels and LIC beyond those in the core study were observed after year 1 of the extension study in both paediatric and adult patients. The decreases were more pronounced in the adult population despite both populations having comparable baseline LIC and serum ferritin, and a similar pattern of response was observed at the end of the core study (9). A number of possible explanations can be considered for the difference between the populations, such as the higher transfusional iron intake (0.36 vs. 0.28 mg/kg/d), the longer median time to first dose increase (40 vs. 26 wk) and the lower mean actual dose received by paediatric patients than by adults. In addition to the clinical practice of more frequent transfusion in paediatric patients, previous pharmacokinetic studies have also shown that systemic exposure to deferasirox is lower in children (13) than in adults (15, 16). The slower rate of dose titration in paediatric patients may be related to investigators using a more conservative approach to dose escalation in this population in the light of limited safety data at the time of study conduct. However, robust safety data have emerged on the use of deferasirox (6, 18–20), including in paediatric patients (21, 22). Taken together, these results highlight the importance of timely dose increases and demonstrate that deferasirox increases to ≥30 mg/kg/d are necessary in heavily iron-overloaded paediatric patients as well as in adults.

At core baseline, mean LIC and median serum ferritin levels were above the thresholds associated with significant negative outcomes (>15 mg Fe/g dw and >2500 ng/mL, respectively) (23, 24), which meant that these patients were at high risk for cardiac complications and death despite the fact that all were previously treated with DFO and/or deferiprone as a monotherapy or combination therapy. This observation suggests that these patients were not adequately chelated before entering the study, perhaps because of issues with compliance, drug response or toxicity. It is encouraging to note that during 2 yr of deferasirox treatment, an increasing proportion of patients achieved LIC <15 mg Fe/g dw (33.9% to 67.8%) and serum ferritin levels <2500 ng/mL (32.6% to 54.2%). This demonstrates that with appropriate dosing over the long term, these higher risk patients can be brought into iron control. Other longer-term studies have shown that with continued treatment, an increasing percentage of patients can achieve iron control within the target range of serum ferritin <2500 ng/mL and/or LIC <7 mg Fe/g dw (21).

Deferasirox was generally well tolerated with a clinically manageable safety profile and a low discontinuation rate during the entire treatment period of this study. The overall safety profile was consistent with previous observations in patients with β-thalassaemia (6, 15, 16) and was generally similar between adult and paediatric patients. The frequency of drug-related AEs decreased in the extension compared with the core study, despite dose increases and a longer median treatment exposure, suggesting that AEs generally occur early in treatment and are mostly transient. Given the low rate of discontinuation observed in this study, this reduction is unlikely to be caused by patients experiencing AEs who discontinued from the trial. Furthermore, there were no progressive increases in serum creatinine or liver transaminase levels over the study period. Indeed, significant decreases in the levels of ALT and AST were observed over the course of the study, indicating improved liver function possibly as a result of hepatic iron chelation. Improved liver function with effective iron chelation has been seen in a number of other deferasirox trials across a variety of underlying anaemia (25–27). Improvement in LVEF within the normal range was observed after 2 yr of deferasirox treatment, suggesting a positive effect of deferasirox on cardiac function, although patients’ myocardial iron status was not evaluated in this study. Data from a separate study showed stabilization of LVEF in patients with mild-to-moderate and severe cardiac siderosis after 2 yr of deferasirox treatment (28).

To assist with treatment compliance, which is essential for effective control of iron overload (29, 30), it is important for physicians to discuss potential AEs with their patients before initiating deferasirox therapy and to reassure patients that if these events do occur then they can be readily managed. Furthermore, the patient should be aware that these events are usually mild and will most often resolve even if they continue to take deferasirox.

Of note, increased deferasirox doses in the paediatric population did not result in any significant increases in AEs. Furthermore, physical development progressed normally during deferasirox treatment. Sexual development also progressed normally according to Tanner stage assessment of patients aged ≥12–<16 yr when compared to a US population, although published data for Middle Eastern children are lacking. This highlights that deferasirox can effectively control body iron burden without any growth-related safety concerns, which is important given that growth retardation and hypogonadism because of iron overload and DFO-induced toxicity are significant clinical problems in paediatric patients with β-thalassaemia (2, 3). It should be noted however that h-SDS are somewhat limited because of comparisons with a non-thalassaemia North American control population. These data from a single study and over longer time horizons are consistent with previous findings from pooled studies highlighting that deferasirox >30 mg/kg/d has a clinically manageable safety profile similar to that seen with doses of <30 mg/kg/d (31).

In conclusion, following treatment with deferasirox, LIC and serum ferritin levels were significantly decreased by 32.4% and 30.2%, respectively, during the initial 2 yr of treatment in these heavily iron-overloaded patients with β-thalassaemia. Doses of ≥30 mg/kg/d were required in most patients because of high transfusional iron intake and heavy iron burden at baseline, highlighting the importance of administering sufficient deferasirox to achieve the clinical goal of net iron reduction. With longer exposure and at doses ≥30 mg/kg/d, deferasirox had a clinically manageable safety profile and was generally well tolerated.

## Appendix

### Participating centres and investigators

Abdullah Al Jefri, King Faisal Specialist Hospital and Research Center, Riyadh, Saudi Arabia; Kusai Al Zir, National Thalassemia Center, Damascus, Syrian Arab Republic; Shahina Daar, Sultan Qaboos University, Muscat, Oman; Mohsen Saleh Elalfy, Ain Shams University, Cairo, Egypt; Amal El-Beshlawy, Cairo University, Cairo, Egypt; Ali Taher, American University of Beirut, Beirut, Lebanon.
